# Ammonium 1-ammonio­ethane-1,1-diylbis(hydrogenphospho­nate) dihydrate

**DOI:** 10.1107/S1600536808037045

**Published:** 2008-11-13

**Authors:** V. V. Bon, A. V. Dudko, A. N. Kozachkova, V. I. Pekhnyo

**Affiliations:** a V. I. Vernadskii Institute of General and Inorganic Chemistry, Kyiv 03680, Ukraine

## Abstract

The title compound, NH_4_^+^·C_2_H_8_NO_6_P_2_^−^·2H_2_O, was obtained by the reaction between 1-amino­ethane-1,1-diyldiphospho­nic acid and ammonium hydroxide (1:1) in an aqueous solution. The asymmetric unit contains one anion with two H atoms transferred from the phospho­nic acid groups to the amino group of the anion and to an ammonia mol­ecule, giving an ammonium cation. The structure displays N—H⋯O and O—H⋯O hydrogen bonding, which creates a three-dimensional network.

## Related literature

Diphospho­nic acids are efficient drugs for the prevention of calcification and the inhibition bone resorption (Tromelin *et al.*, 1986 ▶, Matczak-Jon & Videnova-Adrabinska, 2005 ▶) and are used in the treatment of Pagets disease, osteoporosis and tumoral osteolysis (Szabo *et al.*, 2002 ▶). For related structures, see: Bruckmann *et al.* (1999 ▶); Olive *et al.* (2000 ▶); Coiro *et al.* (1989 ▶). For bond-length data, see: Allen *et al.* (1987 ▶). 
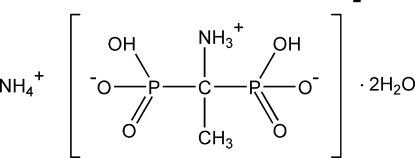


## Experimental

### 

#### Crystal data

NH_4_^+^·C_2_H_8_NO_6_P_2_^−^·2H_2_O
*M* *_r_* = 258.11
Monoclinic, 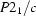

*a* = 8.8922 (3) Å
*b* = 6.9390 (3) Å
*c* = 18.9576 (8) Å
β = 117.957 (2)°
*V* = 1033.23 (7) Å^3^
*Z* = 4
Mo *K*α radiation
μ = 0.45 mm^−1^
*T* = 173 (2) K
0.23 × 0.19 × 0.09 mm

#### Data collection

Bruker SMART APEXII CCD area-detector diffractometer
Absorption correction: multi-scan (*SADABS*; Bruker, 2005 ▶) *T*_min_ = 0.906, *T*_max_ = 0.963
14152 measured reflections
2126 independent reflections
1710 reflections with *I* > 2σ(*I*)
*R*_int_ = 0.057

#### Refinement

*R*[*F* ^2^ > 2σ(*F* ^2^)] = 0.033
*wR*(*F* ^2^) = 0.074
*S* = 1.05
2126 reflections
180 parameters
2 restraints
H atoms treated by a mixture of independent and constrained refinement
Δρ_max_ = 0.50 e Å^−3^
Δρ_min_ = −0.42 e Å^−3^

### 

Data collection: *APEX2* (Bruker, 2005 ▶); cell refinement: *SAINT* (Bruker, 2005 ▶); data reduction: *SAINT*; program(s) used to solve structure: *SHELXTL* (Sheldrick, 2008 ▶); program(s) used to refine structure: *SHELXTL*; molecular graphics: *SHELXTL*; software used to prepare material for publication: *SHELXTL* and *PLATON* (Spek, 2003 ▶).

## Supplementary Material

Crystal structure: contains datablocks I, global. DOI: 10.1107/S1600536808037045/rk2118sup1.cif

Structure factors: contains datablocks I. DOI: 10.1107/S1600536808037045/rk2118Isup2.hkl

Additional supplementary materials:  crystallographic information; 3D view; checkCIF report

## Figures and Tables

**Table 1:** Hydrogen-bond geometry (Å, °)

*D*—H⋯*A*	*D*—H	H⋯*A*	*D*⋯*A*	*D*—H⋯*A*
O3—H3*O*⋯O4^i^	0.78 (3)	1.74 (3)	2.523 (2)	179 (3)
O6—H6*O*⋯O5^ii^	0.81 (3)	1.71 (3)	2.526 (2)	175 (3)
N1—H11*N*⋯O2^iii^	0.94 (3)	1.83 (3)	2.759 (2)	169 (2)
N1—H12*N*⋯O8^i^	0.90 (3)	2.00 (3)	2.873 (3)	164 (2)
N1—H13*N*⋯O3^i^	0.87 (3)	2.08 (3)	2.928 (2)	167 (2)
N2—H21*N*⋯O7	0.88 (3)	2.00 (3)	2.860 (3)	165 (3)
N2—H22*N*⋯O2^iv^	0.85 (3)	2.14 (3)	2.914 (3)	151 (2)
N2—H23*N*⋯O1	0.93 (3)	1.91 (3)	2.832 (3)	171 (3)
N2—H24*N*⋯O1^v^	0.90 (2)	1.97 (3)	2.850 (3)	165 (2)
O7—H71*O*⋯O8	0.83 (3)	1.99 (3)	2.817 (3)	177 (3)
O7—H72*O*⋯O5^vi^	0.80 (3)	1.97 (3)	2.745 (2)	165 (3)
O8—H81*O*⋯O1^vii^	0.768 (17)	2.244 (19)	2.984 (2)	162 (3)
O8—H82*O*⋯O7^viii^	0.775 (18)	1.999 (19)	2.770 (3)	173 (4)

Symmetry codes: (i) 
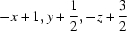
; (ii) 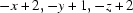; (iii) 
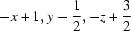
; (iv) 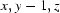; (v) 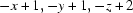; (vi) 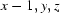; (vii) 
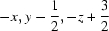
; (viii) 
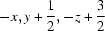
.

**Fig. 1.**
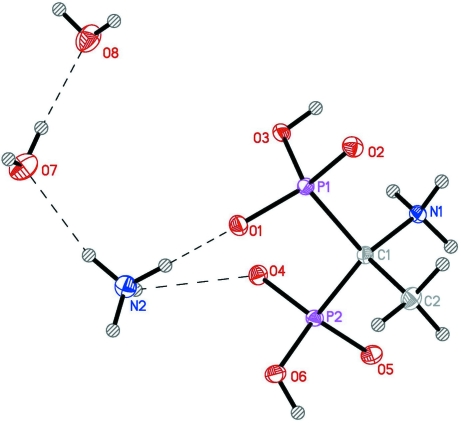
The asymmetric unit of title compound with the atom numbering scheme. The displacement ellipsoids are shown at 50% probability level. H atoms are presented as a small spheres of arbitrary radius.

**Fig. 2.**
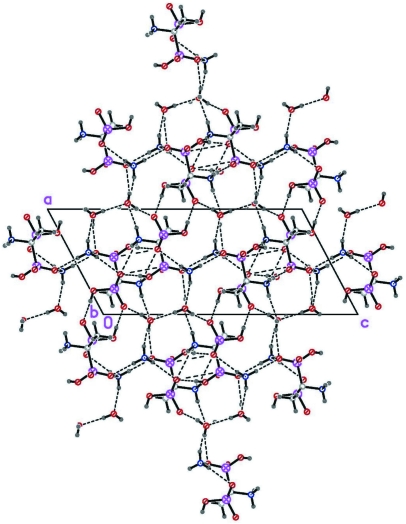
Crystal packing of title compound, projection along b axis. Dashed lines indicate hydrogen bonds.

## supplementary crystallographic information

### Comment

The organic diphosphonic acids are potentially very powerful chelating agents used in metal extractions and are tested by the pharmaceutical industry for use as efficient drugs preventing calcification and inhibiting bone resorption (Tromelin *et al.*, 1986, Matczak-Jon & Videnova-Adrabinska, 2005). Diphosphonic acids are used in the treatment of Paget disease, osteoporosis and tumoral osteolysis (Szabo *et al.*, 2002). The asymmetric unit of title compound (Fig. 1) contains one molecule, which exists as anion with two protons transferred from the phosphonic group to the amino group and from another phosphonic group to ammonium cation. In the crystal structure of the title compound the phosphorus atom displays a slightly distorted tetrahedral geometry provided by three oxygen atoms and one carbon atom (Bruckmann *et al.* (1999); Olive *et al.* (2000); Coiro *et al.* (1989)). Bond lengths and angles have normal values (Allen *et al.*, 1987). One ammonium cation and two solvent water molecules are present in asymetric unit. The structure is stabilized by three-dimensional O–H···O and N–H···O hydrogen bonds network (Table 1, Fig.2).

### Experimental

The title compound was obtained by the reaction of 1-aminoethane-1,1-diyldiphosphonic acid and ammonium hydroxide (1:1) in the aqueous solution. The solution was left at room temperature. Colourless crystals of the title compound were obtained after 1 day staying.

### Refinement

All H atoms bonded to O and N atoms were located in a difference map. Other H atoms bonded to C were positioned geometrically and refined using a riding model with C–H = 0.98 Å for CH_3_ with *U*_iso_(H) = 1.5Ueq(C).

### Crystal data

**Table:**

H_4_N^+^·C_2_H_8_NO_6_P_2_^–^·2H_2_O	*F*_000_ = 544
*M**_r_* = 258.11	*D*_x_ = 1.659 Mg m^−^^3^
Monoclinic, *P*2_1_/*c*	Melting point: 511 K
Hall symbol: -P 2ybc	Mo *K*α radiation λ = 0.71073 Å
*a* = 8.8922 (3) Å	Cell parameters from 4102 reflections
*b* = 6.9390 (3) Å	θ = 2.4–26.4º
*c* = 18.9576 (8) Å	µ = 0.45 mm^−^^1^
β = 117.957 (2)º	*T* = 173 (2) K
*V* = 1033.23 (7) Å^3^	Needle, colourless
*Z* = 4	0.23 × 0.19 × 0.09 mm

### Data collection

**Table:**

Bruker SMART APEXII CCD area-detector diffractometer	2126 independent reflections
Radiation source: Fine-focus sealed tube	1710 reflections with *I* > 2σ(*I*)
Monochromator: Graphite	*R*_int_ = 0.057
*T* = 173(2) K	θ_max_ = 26.5º
φ and ω scans	θ_min_ = 2.4º
Absorption correction: multi-scan(SADABS; Bruker, 2005)	*h* = −11→11
*T*_min_ = 0.906, *T*_max_ = 0.963	*k* = −8→8
14152 measured reflections	*l* = −23→23

### Refinement

**Table:**

Refinement on *F*^2^	Secondary atom site location: Difmap
Least-squares matrix: Full	Hydrogen site location: Geom
*R*[*F*^2^ > 2σ(*F*^2^)] = 0.033	H atoms treated by a mixture of independent and constrained refinement
*wR*(*F*^2^) = 0.074	*w* = 1/[σ^2^(*F*_o_^2^) + (0.0326*P*)^2^ + 0.4969*P*] where *P* = (*F*_o_^2^ + 2*F*_c_^2^)/3
*S* = 1.06	(Δ/σ)_max_ = 0.001
2126 reflections	Δρ_max_ = 0.50 e Å^−^^3^
180 parameters	Δρ_min_ = −0.42 e Å^−^^3^
2 restraints	Extinction correction: None
Primary atom site location: Direct	

### Special details

**Table:**

Geometry. All s.u.'s (except the s.u. in the dihedral angle between two l.s. planes) are estimated using the full covariance matrix. The cell s.u.'s are taken into account individually in the estimation of s.u.'s in distances, angles and torsion angles; correlations between s.u.'s in cell parameters are only used when they are defined by crystal symmetry. An approximate (isotropic) treatment of cell s.u.'s is used for estimating s.u.'s involving l.s. planes.
Refinement. Refinement of *F*^2^ against ALL reflections. The weighted *R*-factor *wR* and goodness of fit *S* are based on *F*^2^, conventional *R*-factors *R* are based on *F*, with *F* set to zero for negative *F*^2^. The threshold expression of *F*^2^ > σ(*F*^2^) is used only for calculating *R*-factors(gt) *etc*. and is not relevant to the choice of reflections for refinement. *R*-factors based on *F*^2^ are statistically about twice as large as those based on *F*, and *R*-factors based on ALL data will be even larger.

### Fractional atomic coordinates and isotropic or equivalent isotropic displacement parameters (Å^2^)

**Table:**

	*x*	*y*	*z*	*U*_iso_*/*U*_eq_	
P1	0.45684 (7)	0.78215 (7)	0.83905 (3)	0.00967 (14)	
P2	0.75545 (7)	0.49061 (7)	0.90274 (3)	0.00998 (14)	
C1	0.6842 (3)	0.7385 (3)	0.86839 (12)	0.0102 (4)	
C2	0.7977 (3)	0.8851 (3)	0.93146 (13)	0.0151 (5)	
H2A	0.9172	0.8604	0.9460	0.023*	
H2B	0.7816	0.8730	0.9790	0.023*	
H2C	0.7668	1.0157	0.9097	0.023*	
N1	0.7091 (2)	0.7652 (3)	0.79539 (11)	0.0110 (4)	
N2	0.4118 (3)	0.2940 (3)	0.92879 (12)	0.0156 (4)	
O1	0.41873 (18)	0.6941 (2)	0.90108 (8)	0.0136 (3)	
O2	0.42601 (18)	0.99358 (19)	0.82470 (8)	0.0138 (3)	
O3	0.35764 (18)	0.6661 (2)	0.75879 (9)	0.0118 (3)	
O4	0.61946 (17)	0.3541 (2)	0.84970 (8)	0.0127 (3)	
O5	0.92422 (18)	0.4672 (2)	0.90282 (8)	0.0137 (3)	
O6	0.7742 (2)	0.4803 (2)	0.98869 (9)	0.0142 (3)	
H3O	0.366 (3)	0.724 (4)	0.7255 (16)	0.033 (8)*	
H6O	0.873 (4)	0.492 (4)	1.0228 (18)	0.044 (9)*	
H11N	0.653 (3)	0.669 (4)	0.7568 (15)	0.022 (6)*	
H12N	0.822 (3)	0.761 (3)	0.8110 (14)	0.016 (6)*	
H13N	0.672 (3)	0.878 (4)	0.7751 (14)	0.019 (7)*	
H21N	0.303 (4)	0.265 (4)	0.9075 (17)	0.036 (8)*	
H22N	0.453 (3)	0.222 (4)	0.9061 (16)	0.028 (8)*	
H23N	0.418 (3)	0.422 (5)	0.9160 (17)	0.039 (8)*	
H24N	0.463 (3)	0.275 (3)	0.9819 (15)	0.013 (6)*	
O7	0.0562 (2)	0.2189 (3)	0.83327 (12)	0.0225 (4)	
O8	−0.0558 (2)	0.3200 (3)	0.67241 (11)	0.0235 (4)	
H71O	0.019 (4)	0.249 (4)	0.786 (2)	0.042 (9)*	
H72O	0.004 (3)	0.278 (4)	0.8504 (16)	0.025 (8)*	
H81O	−0.145 (3)	0.279 (4)	0.6455 (15)	0.034 (9)*	
H82O	−0.064 (4)	0.431 (3)	0.668 (2)	0.063 (12)*	

### Atomic displacement parameters (Å^2^)

**Table:**

	*U*^11^	*U*^22^	*U*^33^	*U*^12^	*U*^13^	*U*^23^
P1	0.0092 (3)	0.0081 (3)	0.0112 (3)	0.0005 (2)	0.0044 (2)	0.0006 (2)
P2	0.0099 (3)	0.0091 (3)	0.0099 (3)	0.0006 (2)	0.0038 (2)	0.0007 (2)
C1	0.0101 (10)	0.0095 (10)	0.0110 (10)	−0.0006 (8)	0.0049 (8)	0.0002 (8)
C2	0.0152 (11)	0.0123 (11)	0.0167 (11)	−0.0028 (9)	0.0065 (9)	−0.0034 (9)
N1	0.0104 (10)	0.0097 (9)	0.0125 (9)	0.0006 (8)	0.0051 (8)	0.0019 (8)
N2	0.0169 (11)	0.0169 (11)	0.0144 (11)	−0.0005 (9)	0.0084 (9)	−0.0014 (8)
O1	0.0138 (8)	0.0141 (8)	0.0144 (8)	0.0004 (6)	0.0078 (6)	0.0025 (6)
O2	0.0155 (8)	0.0104 (7)	0.0152 (7)	0.0010 (6)	0.0069 (6)	0.0004 (6)
O3	0.0130 (8)	0.0103 (7)	0.0108 (7)	−0.0015 (6)	0.0044 (6)	0.0010 (6)
O4	0.0131 (8)	0.0100 (7)	0.0142 (7)	−0.0009 (6)	0.0058 (6)	−0.0007 (6)
O5	0.0117 (8)	0.0144 (8)	0.0141 (7)	0.0018 (6)	0.0052 (6)	−0.0001 (6)
O6	0.0107 (8)	0.0194 (8)	0.0114 (7)	0.0008 (6)	0.0042 (7)	0.0014 (6)
O7	0.0173 (9)	0.0237 (9)	0.0262 (10)	0.0030 (7)	0.0100 (8)	−0.0056 (8)
O8	0.0164 (10)	0.0216 (10)	0.0324 (10)	0.0009 (8)	0.0115 (9)	−0.0013 (8)

### Geometric parameters (Å, °)

**Table:**

P1—O2	1.4939 (14)	N1—H11N	0.94 (3)
P1—O1	1.4982 (14)	N1—H12N	0.90 (3)
P1—O3	1.5760 (15)	N1—H13N	0.87 (3)
P1—C1	1.853 (2)	N2—H21N	0.88 (3)
P2—O4	1.4933 (15)	N2—H22N	0.85 (3)
P2—O5	1.5088 (15)	N2—H23N	0.93 (3)
P2—O6	1.5598 (15)	N2—H24N	0.90 (2)
P2—C1	1.843 (2)	O3—H3O	0.78 (3)
C1—N1	1.512 (3)	O6—H6O	0.81 (3)
C1—C2	1.534 (3)	O7—H71O	0.83 (3)
C2—H2A	0.9800	O7—H72O	0.80 (3)
C2—H2B	0.9800	O8—H81O	0.768 (17)
C2—H2C	0.9800	O8—H82O	0.775 (18)
			
O2—P1—O1	116.99 (8)	H2A—C2—H2B	109.5
O2—P1—O3	110.75 (8)	C1—C2—H2C	109.5
O1—P1—O3	108.61 (9)	H2A—C2—H2C	109.5
O2—P1—C1	107.25 (9)	H2B—C2—H2C	109.5
O1—P1—C1	108.31 (9)	C1—N1—H11N	111.7 (15)
O3—P1—C1	104.13 (9)	C1—N1—H12N	108.2 (15)
O4—P2—O5	115.05 (8)	H11N—N1—H12N	110 (2)
O4—P2—O6	109.31 (8)	C1—N1—H13N	109.0 (15)
O5—P2—O6	112.01 (8)	H11N—N1—H13N	110 (2)
O4—P2—C1	108.55 (9)	H12N—N1—H13N	108 (2)
O5—P2—C1	106.13 (9)	H21N—N2—H22N	106 (3)
O6—P2—C1	105.23 (9)	H21N—N2—H23N	107 (2)
N1—C1—C2	107.87 (16)	H22N—N2—H23N	110 (3)
N1—C1—P2	105.33 (13)	H21N—N2—H24N	110 (2)
C2—C1—P2	110.63 (14)	H22N—N2—H24N	112 (2)
N1—C1—P1	108.22 (14)	H23N—N2—H24N	112 (2)
C2—C1—P1	110.60 (14)	P1—O3—H3O	107 (2)
P2—C1—P1	113.86 (10)	P2—O6—H6O	112 (2)
C1—C2—H2A	109.5	H71O—O7—H72O	108 (3)
C1—C2—H2B	109.5	H81O—O8—H82O	106 (3)
			
O4—P2—C1—N1	−77.19 (14)	O2—P1—C1—N1	−71.57 (14)
O5—P2—C1—N1	47.00 (15)	O1—P1—C1—N1	161.33 (13)
O6—P2—C1—N1	165.88 (13)	O3—P1—C1—N1	45.86 (15)
O4—P2—C1—C2	166.50 (13)	O2—P1—C1—C2	46.40 (16)
O5—P2—C1—C2	−69.31 (15)	O1—P1—C1—C2	−80.71 (15)
O6—P2—C1—C2	49.58 (16)	O3—P1—C1—C2	163.82 (14)
O4—P2—C1—P1	41.22 (13)	O2—P1—C1—P2	171.69 (10)
O5—P2—C1—P1	165.41 (10)	O1—P1—C1—P2	44.58 (13)
O6—P2—C1—P1	−75.71 (12)	O3—P1—C1—P2	−70.88 (12)

### Hydrogen-bond geometry (Å, °)

**Table:**

*D*—H···*A*	*D*—H	H···*A*	*D*···*A*	*D*—H···*A*
O3—H3O···O4^i^	0.78 (3)	1.74 (3)	2.523 (2)	179 (3)
O6—H6O···O5^ii^	0.81 (3)	1.71 (3)	2.526 (2)	175 (3)
N1—H11N···O2^iii^	0.94 (3)	1.83 (3)	2.759 (2)	169 (2)
N1—H12N···O8^i^	0.90 (3)	2.00 (3)	2.873 (3)	164 (2)
N1—H13N···O3^i^	0.87 (3)	2.08 (3)	2.928 (2)	167 (2)
N2—H21N···O7	0.88 (3)	2.00 (3)	2.860 (3)	165 (3)
N2—H22N···O2^iv^	0.85 (3)	2.14 (3)	2.914 (3)	151 (2)
N2—H23N···O1	0.93 (3)	1.91 (3)	2.832 (3)	171 (3)
N2—H24N···O1^v^	0.90 (2)	1.97 (3)	2.850 (3)	165 (2)
O7—H71O···O8	0.83 (3)	1.99 (3)	2.817 (3)	177 (3)
O7—H72O···O5^vi^	0.80 (3)	1.97 (3)	2.745 (2)	165 (3)
O8—H81O···O1^vii^	0.768 (17)	2.244 (19)	2.984 (2)	162 (3)
O8—H82O···O7^viii^	0.775 (18)	1.999 (19)	2.770 (3)	173 (4)

Symmetry codes: (i) −*x*+1, *y*+1/2, −*z*+3/2; (ii) −*x*+2, −*y*+1, −*z*+2; (iii) −*x*+1, *y*−1/2, −*z*+3/2; (iv) *x*, *y*−1, *z*; (v) −*x*+1, −*y*+1, −*z*+2; (vi) *x*−1, *y*, *z*; (vii) −*x*, *y*−1/2, −*z*+3/2; (viii) −*x*, *y*+1/2, −*z*+3/2.
